# Long non-coding RNA MEG3 inhibits NSCLC cells proliferation and induces apoptosis by affecting p53 expression

**DOI:** 10.1186/1471-2407-13-461

**Published:** 2013-10-07

**Authors:** Kai-hua Lu, Wei Li, Xiang-hua Liu, Ming Sun, Mei-ling Zhang, Wei-qin Wu, Wei-ping Xie, Ya-yi Hou

**Affiliations:** 1 Department of Oncology, First Affiliated Hospital, Nanjing Medical University, Nanjing, People’s Republic of China; 2 Department of Biochemistry and Molecular Biology, Nanjing Medical University, Nanjing, People’s Republic of China; 3 Department of respiratory, First Affiliated Hospital, Nanjing Medical University, Nanjing, People’s Republic of China; 4 Immunology and Reproductive Biology Lab of Medical School and State Key Laboratory of Pharmaceutical Biotechnology, Nanjing University, Nanjing, People’s Republic of China

**Keywords:** Long non-coding RNA, *MEG3*, NSCLC, Proliferation, p53

## Abstract

**Background:**

Long non-coding RNAs play an important role in tumorigenesis, hence, identification of cancer-associated lncRNAs and investigation of their biological functions and molecular mechanisms are important for understanding the development and progression of cancer. Recently, the downregulation of lncRNA MEG3 has been observed in various human cancers. However, its role in non-small cell lung cancer (NSCLC) is unknown. The aim of this study was to examine the expression pattern of MEG3 in NSCLC and to evaluate its biological role and clinical significance in tumor progression.

**Methods:**

Expression of MEG3 was analyzed in 44 NSCLC tissues and 7 NSCLC cell lines by qRT-PCR. Over-expression approaches were used to investigate the biological functions of MEG3 in NSCLC cells. Bisulfite sequencing was used to investigate DNA methylation on MEG3 expression. The effect of MEG3 on proliferation was evaluated by MTT and colony formation assays, and cell apoptosis was evaluated by Hoechst staining and Flow-cytometric analysis. NSCLC cells transfected with pCDNA-MEG3 were injection into nude mice to study the effect of MEG3 on tumorigenesis in *vivo* . Protein levels of MEG3 targets were determined by western blot analysis. Differences between groups were tested for significance using Student’s *t*-test (two-tailed).

**Results:**

*MEG3* expression was decreased in non-small cell lung cancer (NSCLC) tumor tissues compared with normal tissues, and associated with advanced pathologic stage, and tumor size. Moreover, patients with lower levels of MEG3 expression had a relatively poor prognosis. Overexpression of *MEG3* decreased NSCLC cells proliferation and induced apoptosis *in vitro* and impeded tumorigenesis *in vivo*. MDM2 and p53 protein levels were affected by MEG3 over-expression *in vitro*.

**Conclusions:**

Our findings indicate that MEG3 is significantly down-regulated in NSCLC tissues that could be affected by DNA methylation, and regulates NSCLC cell proliferation and apoptosis, partially via the activition of p53. Thus, MEG3 may represent a new marker of poor prognosis and is a potential therapeutic target for NSCLC intervention.

## Background

Non-small cell lung cancer (NSCLC) including adenocarcinoma and squamous cell carcinoma, is a predominant form of lung cancer, and accounts for the majority of lung cancer associated deaths worldwide [1]. Despite the recent advances in clinical and experimental oncology, the prognosis of lung cancer is still unfavorable, with a 5-year overall survival rate of approximately 11% [2]. Thus, a detailed understanding of the mechanisms underlying NSCLC development and progression is essential for improving diagnosis, prevention and treatment of this disease. Recently, there is growing evidence indicating that non-coding RNAs may be involved in NSCLC pathogenesis, providing new insights into the biology of this disease [3,4].

Recent improvements in high-throughput transcriptome analysis in the last few years, have led to the discovery that > 90% of the total mammalian genome can be transcribed and may yield many short or long non-coding RNAs (lncRNAs) with limited or no protein-coding capacity [5,6]. Although many studies have helped unraveling the function of microRNAs, the lncRNAs counterpart of the transcriptome is less well characterized. lncRNAs are known to play important roles during cellular development and differentiation, and a large range of functions, such as modulation of proliferation and invasiveness of tumors [7], and reprogramming of induced pluripotent stem cells [8] have been attributed to lncRNAs. Dysregulation of some lncRNAs has been shown in various types of cancers, such as breast cancer, hepatocellular carcinoma, melanoma, bladder cancer, and prostate cancer [7,9-14]. One such lncRNA, HOTAIR, has been determined as a negative prognostic indicator in breast, liver and pancreatic cancer patient survival, evidencing a close association with breast cancer cell metastasis [7,15,16]. Recent studies have also revealed the contribution of lncRNAs, as proto-oncogenes (e.g. *ANRIL*) and tumor suppressor genes (e.g. *MEG3*) in tumorigenesis [17,18].

*Maternally expressed gene 3* (*MEG3*), an lncRNA, is expressed in many normal tissues. However, *MEG3* expression is lost in an expanding list of primary human tumors, and promoter hypermethylation or hypermethylation of the intergenic differentially methylated region has been shown to contribute to the loss of *MEG3* expression in tumors [19,20]. *MEG3* represents as a tumor suppressor gene, and its ectopic expression can inhibit cell proliferation and promote cell apoptosis in human glioma cell lines [21]. Moreover, accumulation of p53 (TP53) protein and its target gene expression partly contribute to cell growth inhibition induced by *MEG3*[22]. However, very little is known about *MEG3* expression level in NSCLC, and its role in NSCLC development.

In this study, we demonstrated that *MEG3* expression was significantly decreased in NSCLC tissues compared to adjacent normal tissues. The correlation between *MEG3* downregulation and advanced pathologic stage, tumor size, and patient survival time was also explored. Moreover, ectopic expression of *MEG3* inhibited cell proliferation and promoted cell apoptosis in human NSCLC cell lines and overexpression of *MEG3* was able to impede the development of tumors *in vivo*. We further verified that overexpression of *MEG3* could induce the activation of p53. Taken together, this study indicated that lncRNA, especially *MEG3* plays an important role in NSCLC development and could be a potential therapeutic target for patients with NSCLC.

## Methods

### Patient and tissue samples

Paired NSCLC and adjacent non-tumor lung tissues were obtained from 44 patients who underwent primary surgical resection of NSCLC between 2006 and 2007 at First Affiliated Hospital of Nanjing Medical University, China. NSCLC and normal tissues were immediately snap-frozen in liquid nitrogen and stored at −80°C until total RNA was extracted. Tumor samples were at least 80% composed of viable-appearing tumor cells on histological assessment. The pathological stage, grade and nodal status were appraised by an experienced pathologist. Clinicopathologic characteristics including tumor-node-metastasis (TNM) staging were also collected. The study was approved by the Research Ethics Committee of Nanjing Medical University, China. Informed written consents were obtained from all patients who participated in this study.

### Cell lines and culture conditions

Six NSCLC adenocarcinoma cell lines (A549, SPC-A1, NCI-H1650, NCI-H358, NCI-H1299, NCI-H1975), a NSCLC squamous carcinomas cell line (SK-MES-1), and a normal human bronchial epithelial cell line (16HBE) were purchased from the Institute of Biochemistry and Cell Biology of the Chinese Academy of Sciences (Shanghai, China). 16HBE, A549, NCI-H1650, NCI-H358, NCI-H1975 and NCI-H1299 cells were cultured in RPMI 1640 medium; SPC-A1, and SK-MES-1 cells were cultured in DMEM (GIBCO-BRL) medium, supplemented with 10% fetal bovine serum (10% FBS), 100 U/ml penicillin, and 100 mg/ml streptomycin (Invitrogen, Shanghai, China) in humidified air at 37°C with 5% CO_2_.

### RNA extraction and qRT-PCR analysis

Total RNA was isolated with TRIzol reagent (Invitrogen, Carlsbad, CA, USA) according to the manufacturer’s protocol. 500 ng total RNA was reverse transcribed in a final volume of 10 μl using random primers under standard conditions using the PrimeScript RT reagent Kit. Assays were performed to detect *MEG3* expression using the PrimeScript RT reagent Kit and SYBR Premix Ex Taq (TaKaRa, Dalian, China) according to the manufacturer’s instructions.

The relative levels of *MEG3* were determined by qPCR using gene specific primers. *GAPDH* was measured as an internal control, as its expression showed minimal variation in different cell lines and cancer specimens. The RT reaction was carried out under the following conditions: 37°C for 15 min; 85°C for 5 sec; and then held on 4°C. After the RT reaction, 1ul of the complementary DNA was used for subsequent qRT-PCR reactions. The PCR primers for *MEG3* or *GAPDH* were as follows: *MEG3* sense, 5′ CTGCCCATCTACACCTCACG 3′ and reverse, 5′ CTCTCCGCCGTCTGCGCTAGGGGCT 3′; *GAPDH* sense, 5′ GTCAACGGATTTGGTCT GTATT 3′ and reverse, 5′ AGTCTTCTGGGTGGCAGTGAT 3′. The PCR reaction was conducted at 95°C for 30 s and followed by 40 cycles of 95°C for 5 s and 60°C for 34 s in the ABI 7500 real-time PCR system (Applied Biosystems, Foster City, CA, USA). The qPCR results were analyzed and expressed as relative mRNA expression of CT (threshold cycle) value, which was then converted to fold changes.

### Methylation analysis of CpG island

For determination of methylation status of the CpG island, genomic DNA prepared from NSCLC cells and normal tissues, was modified by sodium bisulfite (EZ DNA Methylation Kit , Zymo Research), followed by PCR using the sense primer 5′ TTTTTTTGTTGTAATTTGGGTG 3′ and reverse, 5′ ACGAATACCGTCTTCCTTTTAC 3′, respectively. PCR-amplified product was transformed in E.coli DH5α cells. Subsequently obtained plasmids were subjected to sequencing.

### Treatment of SPC-A1 cells with 5-aza-2-deoxy-cytidine (5-aza-CdR)

SPC-A1 cells (2.5 × 10^5^) were seeded into six-well culture plate on day 0 and exposed to 0, 2 or 5 μM 5-aza-CdR(Sigma-Aldrich, USA)from day 1 to day 3. The cells treated with 5-aza-CdR were harvested on day 3 and used for detection of *MEG3* expression.

### Plasmid constructs

The sequence of *MEG3* was synthesized and subcloned into pCDNA3.1 (Invitrogen, Shanghai, China). Ectopic expression of *MEG3* was achieved by using the pCDNA-MEG3 transfection and empty pCDNA vector (empty) was used as control. The expression level of *MEG3* was detected by qPCR.

### Transfection of NCSCL cells

All plasmid vectors (pCDNA-MEG3 and empty vector) for transfection were extracted by DNA Midiprep or Midiprep kit (Qiagen, Hilden, Germany). SPC-A1 and A549 cells cultured on six-well plate were transfected with the pCDNA -MEG3 or empty vector using Lipofectamine2000 (Invitrogen, Shanghai, China) according to the manufacturer’s instructions. Cells were harvested after 48 hours for qRT-PCR and western blot analyses.

### Cell proliferation assays

Cell proliferation was monitored using Cell Proliferation Reagent Kit I (MTT) (Roche Applied Science). pCDNA-MEG3 and empty vector transfected SPC-A1 cells (3000/well) were allowed to grow in 96-well plates. Cell proliferation was measured every 24 hours following the manufacturer’s protocol. All experiments were performed in quadruplicate. For colony formation assay, a total of 500 pCDNA-MEG3 and empty vector cells were placed in a fresh six-well plate and maintained in media containing 10% FBS, replacing the medium every 4 days. After 14 days, cells were fixed with methanol and stained with 0.1% crystal violet (Sigma-Aldrich (country???)). Visible colonies were manually counted. Triplicate wells were measured for each treatment group.

### Flow-cytometric analysis of apoptosis

SPC-A1 and A549cells transfected with pCDNA-MEG3 and empty vector were harvested 48 hours after transfection by trypsinization. Following double staining with FITC-Annexin V and Propidium iodide (PI), the cells were analyzed using flow cytometry (FACScan®; BD Biosciences) equipped with a CellQuest software (BD Biosciences) [23]. Cells were discriminated into viable cells, dead cells, early apoptotic cells, and apoptotic cells. The percentage of early apoptotic cells were compared to control groups from each experiment. All of the samples assayed were in triplicates.

### Hoechst staining assay

SPC-A1 and A549 cells transfected with pCDNA-MEG3 and empty vector were cultured in six-well plates, and were incubated with Hoechst 33342 solution (50 ng/ml, Sigma-Aldrich, St Louis, MO, USA) for 10 min at room temperature. Cells were then washed twice with PBS and changes in nuclear morphology were detected by fluorescence microscopy using 365 nm filter for Hoechst 33342. For quantification of Hoechst 33342 staining, the percentage of Hoechst -positive nuclei per optical field (at least 50 fields) was counted in three independent experiments.

### Tumor formation assay in a nude mouse model

Female athymic BALB/c nude mice aged 4 weeks were maintained under specific pathogen-free conditions and manipulated according to protocols approved by the Shanghai Medical Experimental Animal Care Commission. SPC-A1 cells were transfected with pCDNA-MEG3 and empty vector and harvested from six-well cell culture plates, washed with PBS, and resuspended at a concentration of 2 × 10^7^ cells/mL. A volume of 0.1 mL of suspended cells was subcutaneously injected into a single side of the posterior flank of each mouse. Tumor growth was examined every three days, and tumor volumes were calculated using the equation V = 0.5 × D × d^2^ (V, volume; D, longitudinal diameter; d, latitudinal diameter) [16]. At 3 weeks post injection, mice were euthanized, and the subcutaneous growth of each tumor was examined.

This study was carried out in strict accordance with the recommendations in the Guide for the Care and Use of Laboratory Animals of the National Institutes of Health. The protocol was approved by the Committee on the Ethics of Animal Experiments of the Nanjing medical University (Permit Number: 200933). All surgery was performed under sodium pentobarbital anesthesia, and all efforts were made to minimize suffering in mice [24].

### Western blotting assay

Cells were lysed using mammalian protein extraction reagent RIPA (Beyotime china) supplemented with protease inhibitors cocktail (Roche. Switzerland) and PMSF (Roche, Switzerland). Protein concentration was measured with the Bio-Rad protein assay kit. 50 μg protein extractions were separated by 12% SDS-polyacrylamide gel electrophoresis (SDS-PAGE), then transferred to 0.22 μm nitrocellulose membranes (Sigma-Aldrich. USA)and incubated with specific antibodies. ECL chromogenic substrate was used to visualize the bands and the intensity of the bands was quantified by densitometry (Quantity One software; Bio-Rad). GAPDH was used as control. GAPDH antibody was purchased from sigma-Aldrich (USA), P53 antibody was purchased from Santa Cruz Biotechnology (Santa Cruz, CA, USA), P21 antibody was purchased from Cell Signaling Technology (MA, USA).

### Statistical Analysis

Student’s *t*-test (two-tailed), One-way ANOVA and Mann–Whitney test were performed to analyze the data using SPSS 16.0 software. *P* values less than 0.05 were considered statistically significant.

## Results

### *MEG3* expression is downregulated in human NSCLC tissues

**Figure 1:**
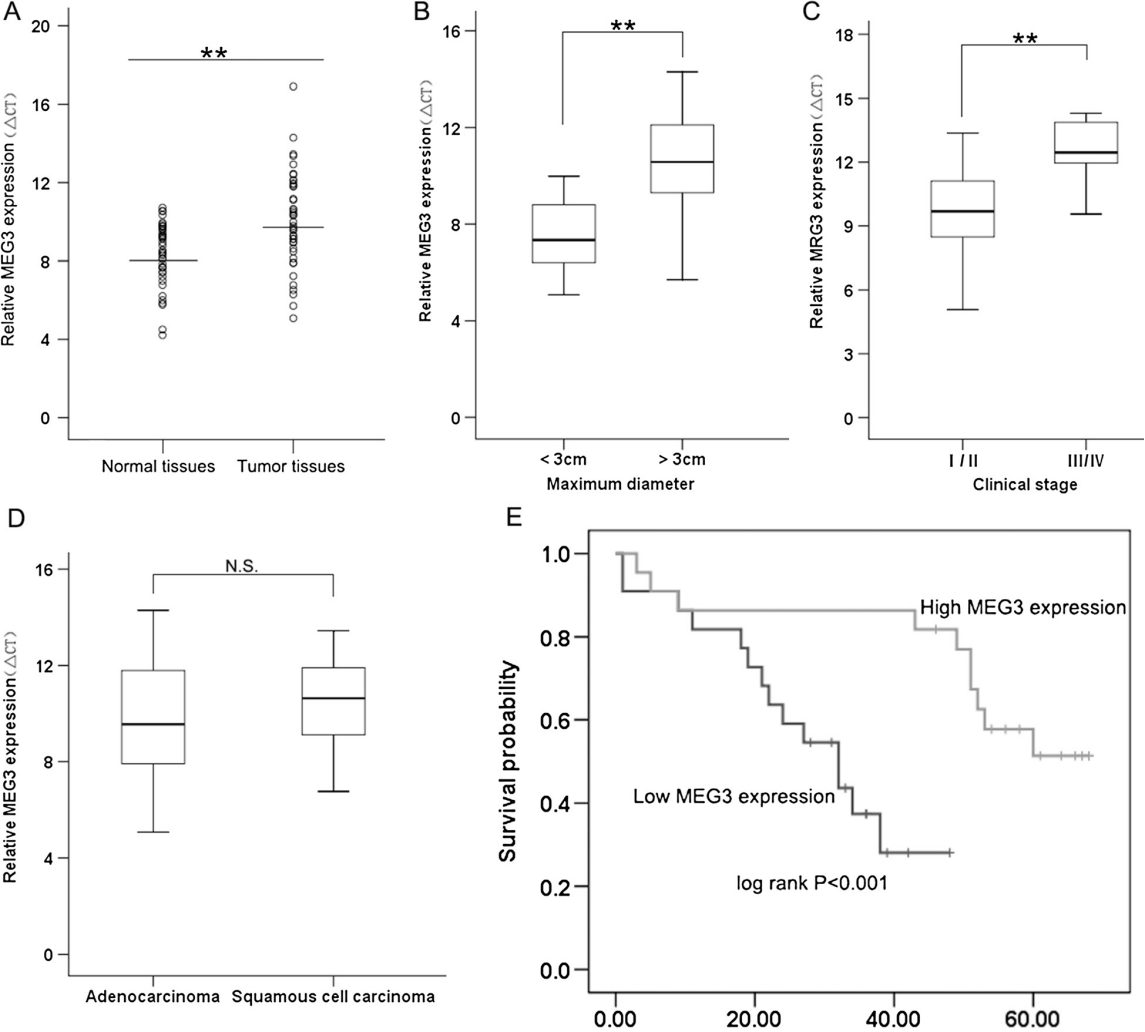
**qRT-PCR analysis of lncRNA MEG3 in NSCLC tissues. (A) ***MEG3* expression in NSCLC tissues and its clinical significance. *MEG3* was measured in 44 pair NSCLC and normal tissues by qRT-PCR (shown as ΔCT). (**B** and **C**) Data are presented as relative expression level in tumor tissues (shown as ΔCT). *MEG3* expression was significantly lower in patients with a higher pathological stage and big tumor size. **(D)** Patients with low levels of *MEG3* expression showed reduced survival times compared to patients with high levels of *MEG3* expression (log rank *P* < 0.001). **, *P* < 0.01.

qRT–PCR analysis was used to measure *MEG3* expression in 44 NSCLC tissues and normal counterparts. The expression of *MEG3* was significantly downregulated in NSCLC tissues (Figure 1A). Furthermore, correlation analysis of *MEG3* expression with clinical pathological features of NSCLC patients, revealed a significant association between *MEG3* downregulation and advanced pathological stage (I/II,37; IIIa/b,IV,7) and NSCLC tumor size (Figure 1B,C). However, *MEG3* expression was not correlated with histological subtype, patient age, gender, or tumor position (Figure 1D and Table 1). Clinical data of individual patients is shown in Additional file 1: Table S1.

**Table 1:** Correlation of the expression of MEG3 with clinicopathologic features

**Clinicopathologic features**	**n (%)**	**Relative expression of MEG3**^**a**^	**P-value**^**b**^
Gender			P = 0.653
Male	34 (77)	0.36	
Female	10 (23)	0.42	
Site of tumor			P = 0.758
Left lung	19 (43)	0.31	
Right lung	25 (57)	0.39	
Differentiation			P = 0.073
Poor	16 (36)	0.27	
Moderate	28 (64)	0.45	
Lymph node metastasis			P = 0.042
Yes	27 (61)	0.42	
No	17 (39)	0.64	

Correlation of the expression of MEG3 with clinicopathologic features. a Median of relative expression. b P < 0.05 was considered significant (Mann–Whitney *U* test between 2 groups and Kruskall-Wallis test for 3 groups).

Kaplan-Meier survival analysis and log-rank tests using patient postoperative survival were performed to further evaluate the correlation between *MEG3* expression and NSCLC patient prognosis. According to the median ratio of relative *MEG3* expression (0.27) in tumor tissues, the 44 NSCLC patients were classified into two groups: High-MEG3 group (*n* = 21, *MEG3* expression ratio ≥ mean ratio) and Low-MEG3 group (*n* = 21, *MEG3* expression ratio ≤ mean ratio). The Kaplan-Meier survival curve showed that patients with decreased *MEG3* expression levels had significantly shorter survival times than those with high *MEG3* expression levels (Figure 1D). These findings support the hypothesis that decreased *MEG3* expression plays a key role in NSCLC development and progression.

### Effect of DNA methylation on *MEG3* expression

**Figure 2:**
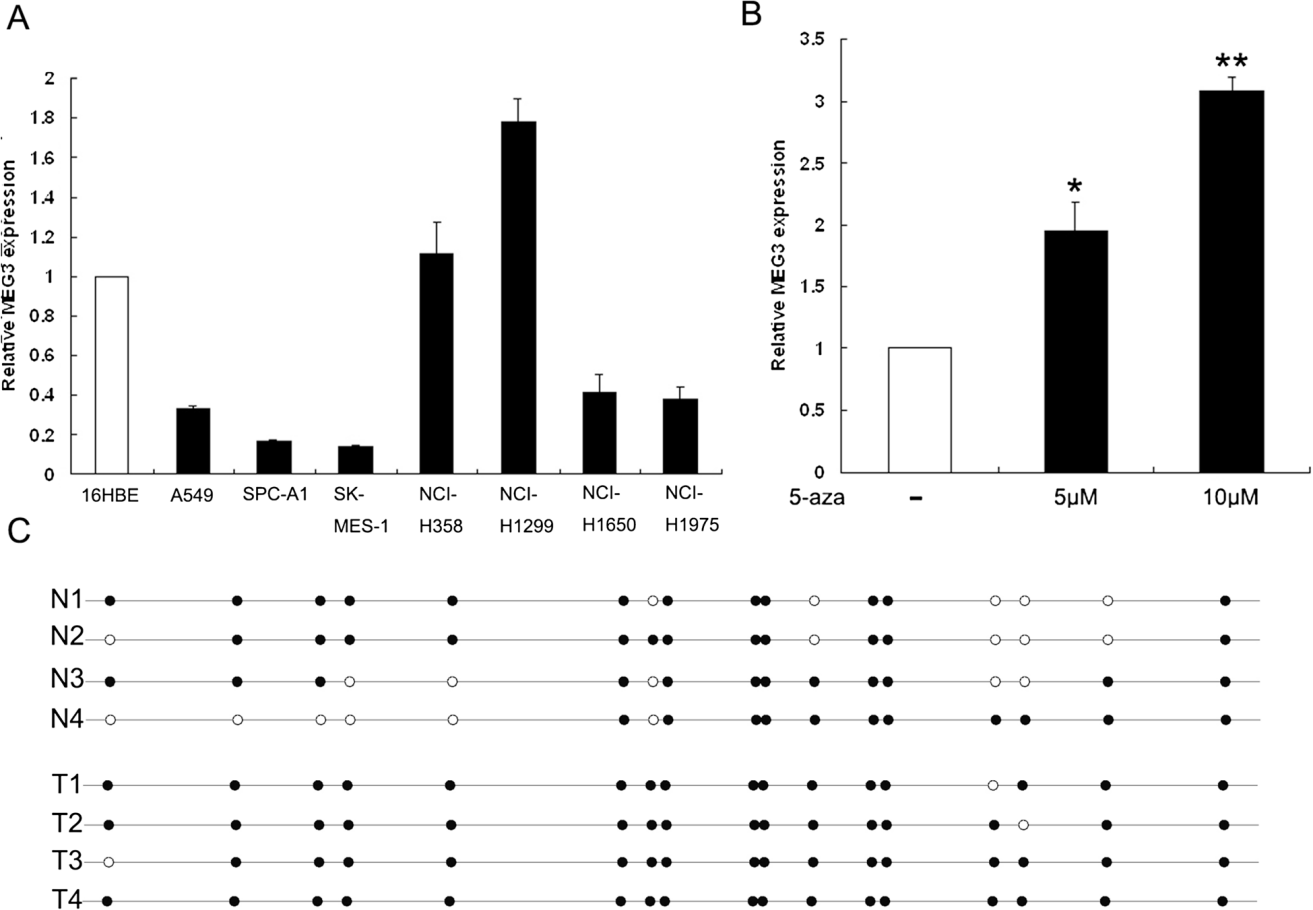
**Analysis of the correlation between methylation status and expression of *MEG3*. (A)** Analysis of *MEG3* expression levels in NSCLC cell lines (A549, SPC-A1, NCI-H1650, NCI-H1299, NCI-358, NCI-H1975 and SK-MES-1) compared with the normal bronchial epithelial cell line (16HBE) by qRT-PCR. **(B)** The level of *MEG3* expression in SPC-A1 cells following 5-aza-dC (0, 5, 10 μM) treatment. **(C)** The methylation status of the CpG island of *MEG3* was assessed by bisulfite sequencing in NSCLC and normal tissues. Open and filled squares denote unmethylated and methylated CpG sites, respectively. Each row represents a single clone. *P < 0.05; **P < 0.01.

We next performed qRT-PCR analysis to examine the expression of *MEG3* in 7 human NSCLC cell lines, including both adenocarcinoma and squamous carcinoma subtypes. Of these, five cell lines (A549, SPC-A1, NCI-H1650, NCI-H1975 and SK-MES-1) expressed lower levels of *MEG3* compared with the normal bronchial epithelial cell line and 16HBE, while NCI-H358 and H1299 cells expressed relatively higher endogenous levels of *MEG3* (Figure 2A). The expression of lncRNA is more cell sepecific [25], which may contribute to the different expression level of MEG3 in NSCLC cell lines.

The expression of *MEG3* was frequently downregulated in NSCLC, and hypermethylation of MEG3-MDR has been reported to be involved in *MEG3* transcriptional inactivation. Following treatment of SPC-A1 cells with DNA demethylating agent (5-aza-CdR), we found that *MEG3* expression was significantly increased by 1.95- or 3.08-fold in 5-aza-CdR treated cells compared with control (Figure 2B). Moreover, among the three canonical CpG island of *MEG3-DMR* loci (DMR1, DMR2 and DMR3), we examined the methylation pattern of DMR2 in NSCLC and normal tissues by bisulfite sequencing, and the average frequency of methylation was 68% in normal tissues and 96% in NSCLC tissues (Figure 2C). These results indicate that downregulation of *MEG3* observed in NSCLC cells might have been partly due to hypermethylation of *MEG3-DMR*.

### Effect of *MEG3* on cell proliferation *in vitro*

**Figure 3:**
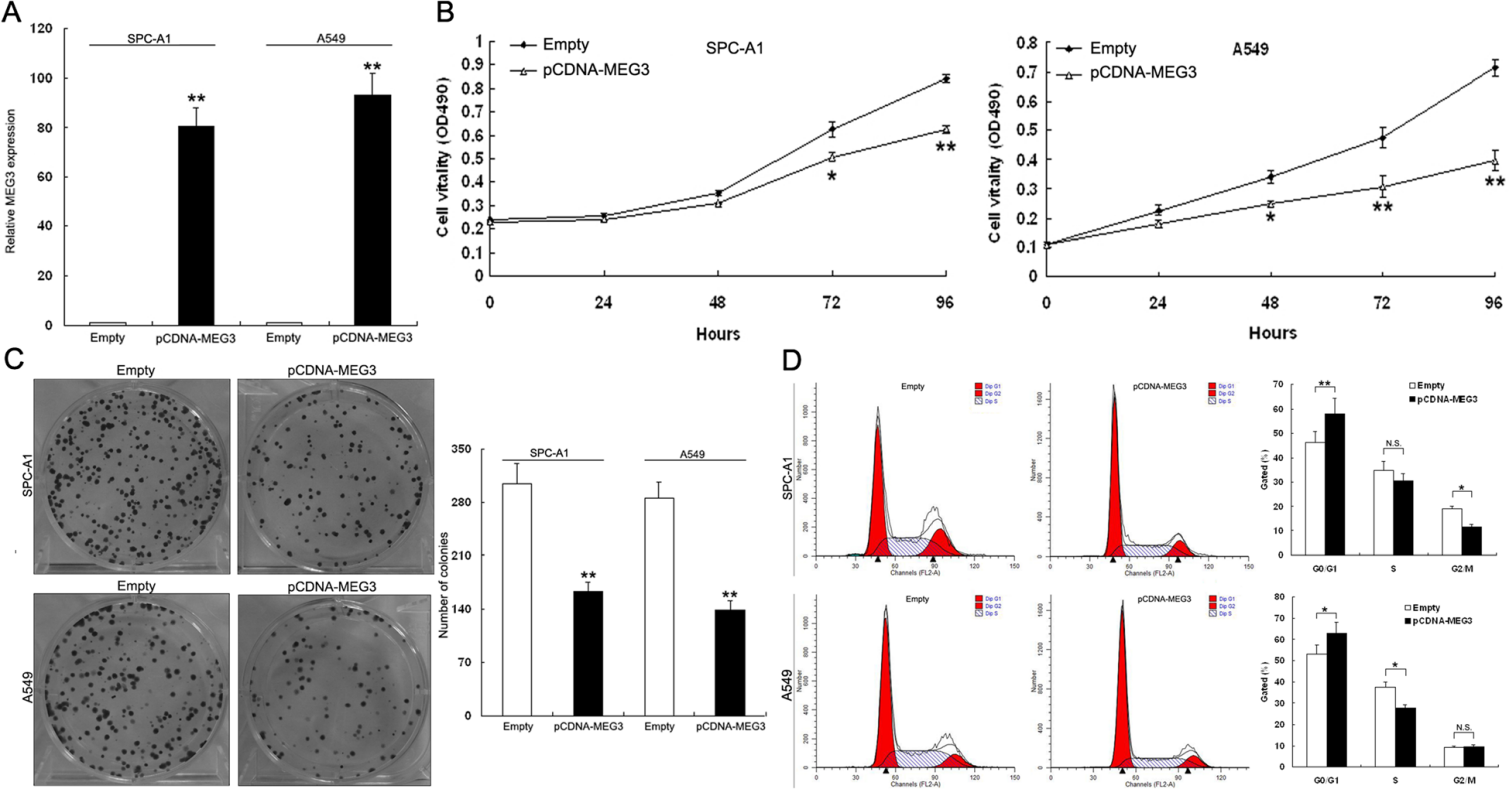
**Effects of *MEG3 *on cell proliferation *in vitro*. (A)** Analysis of *MEG3* expression levels in SPC-A1 and A549 cells transfected with PCDNA-MEG3 or empty vector by qRT-PCR. **(B)** MTT assay was performed to determine the proliferation of SPC-A1 and A549 cells. Data represent the mean ± S.D. from three independent experiments. **(C)** Colony-forming growth assays were performed to determine the proliferation of SPC-A1 and A549 cells. The colonies were counted and captured. **(D)** The bar chart represented the percentage of cells in G0/G1, S, or G2/M phase, as indicated. All experiments were performed in biological triplicates with three technical replicates.*P < 0.05, **P < 0.01.

*MEG3 was* overexpressed in SPC-A1 and A549 cells by transfecting them with pCDNA-MEG3. qRT-PCR analysis of *MEG3* levels revealed that *MEG3* expression was increased by 80-fold or 91-fold in SPC-A1 or A549 cells respectively following transfection with pCDNA-MEG3 compared with control (Figure 3A).

To assess the biological role of *MEG3* in NSCLC, we investigated the effects of targeted overexpression of *MEG3* on cell proliferation. MTT assay revealed that cell growth was significantly impaired in SPC-A1 and A549 cells transfected with pCDNA-MEG3 compared with controls (Figure 3B). Similarly, the results of colony-formation assays revealed that clonogenic survival was decreased following enhanced *MEG3* expression in SPC-A1 and A549 cells (Figure 3C). To further examine whether the effect of *MEG3* on proliferation of NSCLC cells was on cell cycle regulation, cell cycle progression was analyzed by flow cytometry. The results revealed that SPC-A1 and A549 cells transfected with pCDNA-MEG3 had an obvious cell cycle arrest at the G1/G0 phase and had a decreased G2/S phase (Figure 3D). Moreover, inhibition of *MEG3* expression in H1299 cells promoted cells proliferation (Additional file 2: Figure S1)

### Effect of *MEG3* on cell apoptosis and invasion

**Figure 4:**
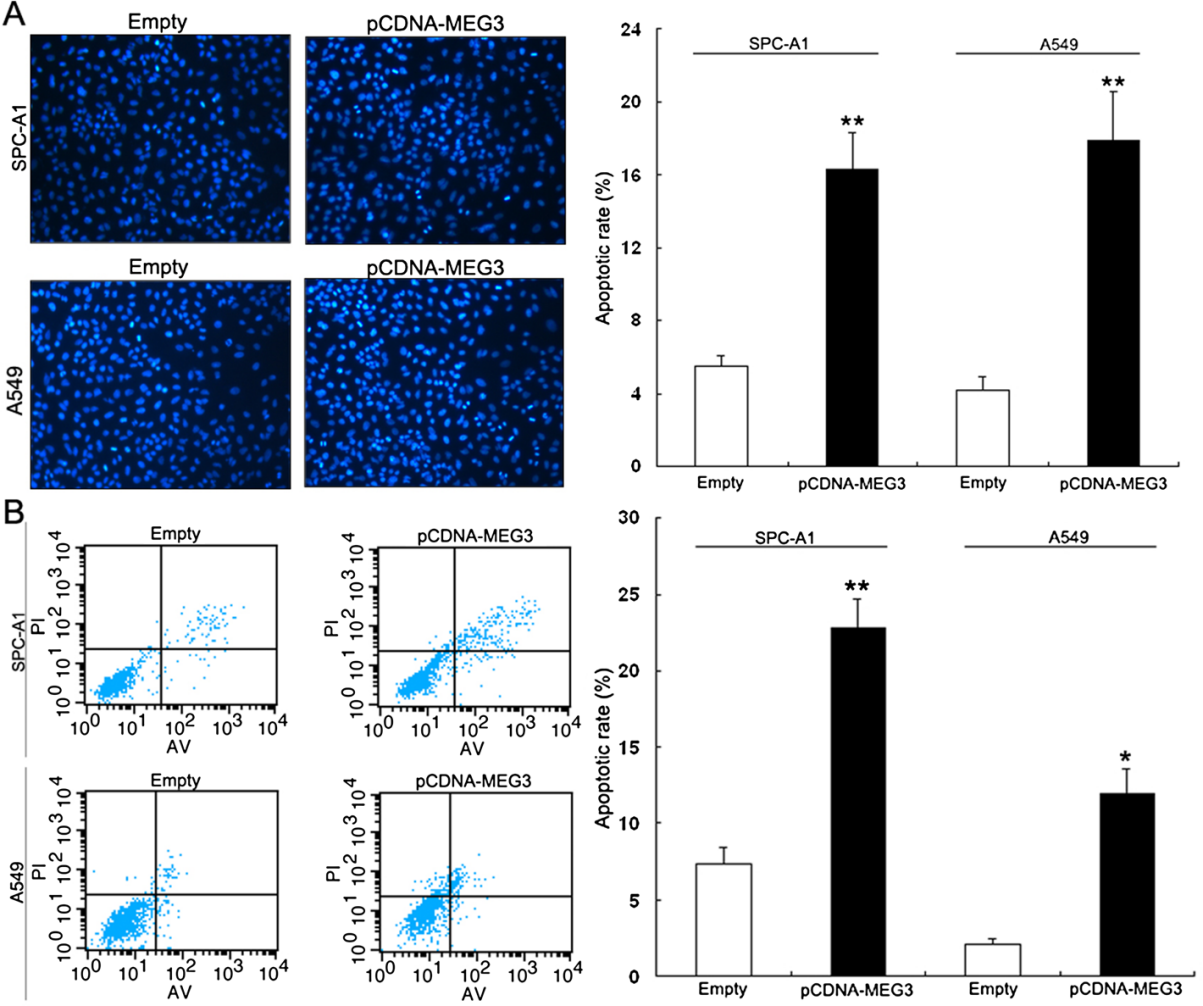
**Effects of *MEG3 *on cell apoptosis. (A)** Hoechst staining assay for cell apoptosis; the percentage of Hoechst-positive nuclei per optical field (at least 50 fields) was counted. **(B)** The apoptotic rates of cells were detected by flow cytometry. *P < 0.05 and **P < 0.01.

To determine whether apoptosis was a contributing factor to cell growth inhibition, we performed Hochest staining and flow-cytometric analysis after transfection with pCDNA-MEG3. The apoptotic rate of SPC-A1 and A549 cells transfected with pCDNA-MEG3 increased by approximately 11% and 12% respectively in comparison with cells transfected with empty vector (Figure 4A,B).

Cell invasion is a significant aspect of cancer progression, and involves the migration of tumor cells into contiguous tissues and the dissolution of extracellular matrix proteins. To investigate whether *MEG3* had a direct functional role in facilitating cell invasion in NSCLC, we evaluated cancer cell invasion through transwell matrigel assay. However, alteration of *MEG3* expression had no significant effects on cell invasion compared with control (data not shown).

### *MEG3* inhibits tumorigenesis of NSCLC cells *in vivo*

**Figure 5:**
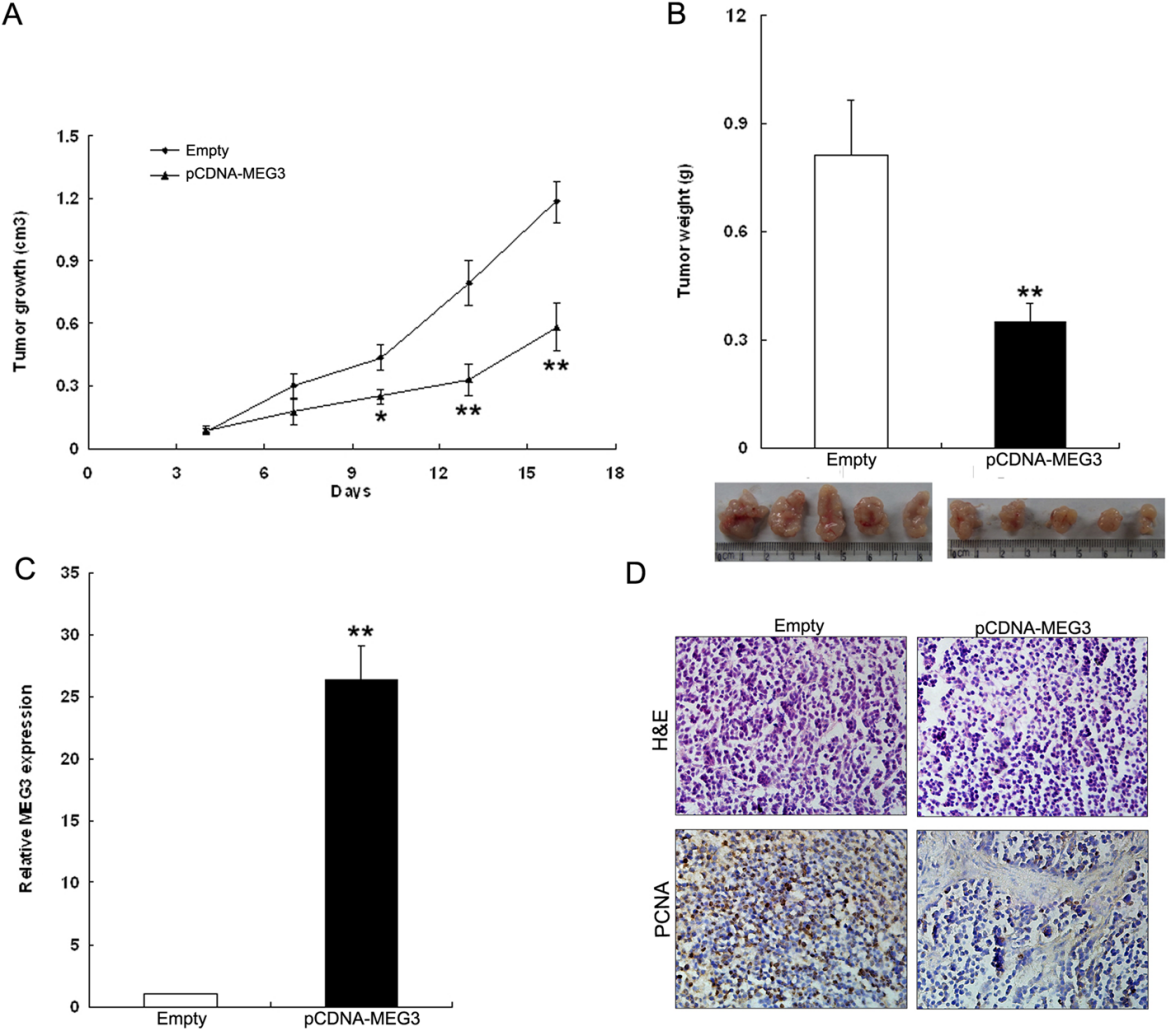
**Effects of *MEG3 *on tumor growth *in vivo*. (A)** The tumor volume was calculated once every three days after injection of SPC-A1 cells stably transfected with PCDNA-MEG3 or empty vector. Points, mean (n = 3); bars indicate S.D. **(B)** Tumor weights are represented as means of tumor weights ± s.d. **(C)** QPCR analysis of *MEG3* expression in tumor tissues formed from SPC-A1/MEG3, SPC-A1/NC. **(D)**. Tumors developed from PCDNA-MEG3 transfected SPC-A1 cells showed lower PCNA protein levels than tumors developed by control cells. Upper: H & E staining; Lower: immunostaining. *P < 0.05, **P < 0.01 and N.S. not significant.

To explore whether the level of *MEG3* expression affects tumorigenesis, pCDNA-MEG3 and empty vector stably-transfected SPC-A1 cells were inoculated into female nude mice. Eighteen days after injection, the tumors formed in pCDNA-MEG3 group were substantially smaller than those in the empty vector group (Figure 5A). Moreover, the mean tumor weight at the end of the experiment was markedly lower in the pCDNA-MEG3 group (0.35 ± 0.11 g) compared to the control group (0.81 ±0.15 g) (Figure 5B). qRT-PCR analysis of *MEG3* expression was then performed in selected tumor tissues. The results showed that the levels of *MEG3* expression in tumor tissues formed from pCDNA-MEG3 cells were higher than those of tumors formed in control group (Figure 5C). Immunostaining was used to analyze PCNA protein expression in resected tumor tissues. PCNA levels in tumors formed from control cells (empty vector), exhibited decreased positivity for PCNA than in tumors from pCDNA-MEG3 transfected SPC-A1 cells (Figure 5D). These results indicate that overexpression of *MEG3* could inhibit tumor growth *in vivo*.

### *MEG3* stimulates activation of p53 protein

**Figure 6:**
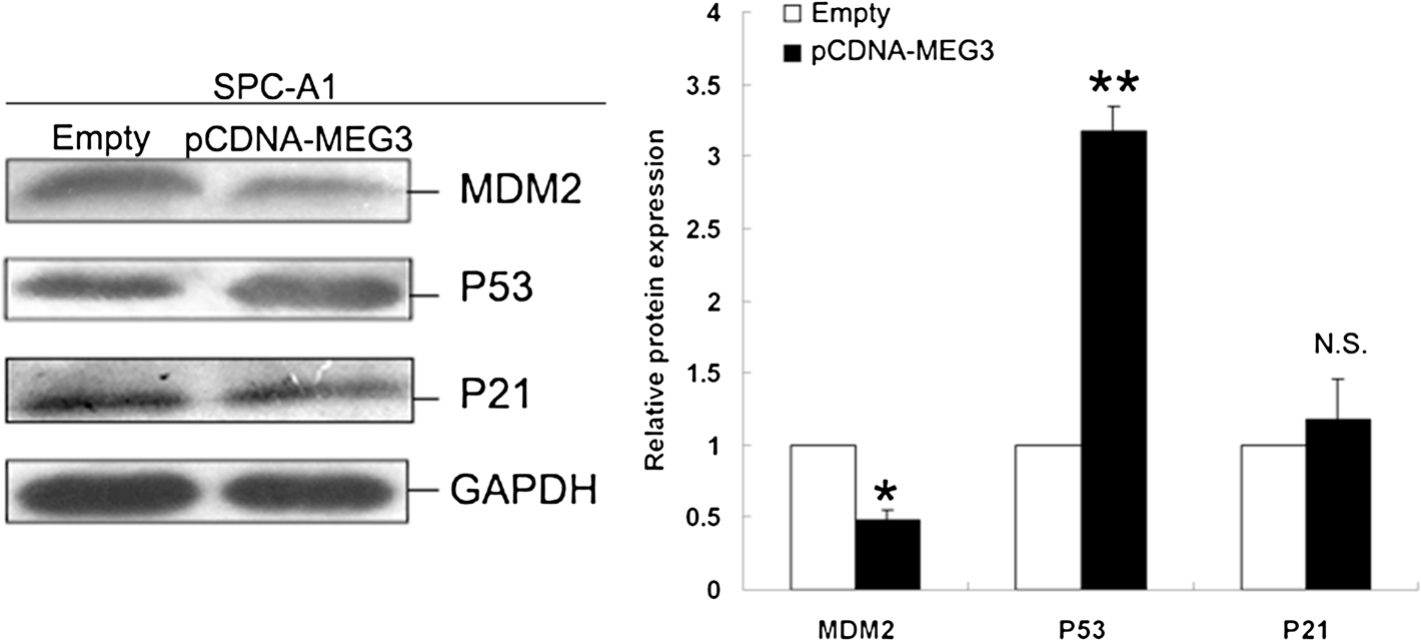
***MEG3 *increased p53 activation. Western blot analysis of p53, MDM2 and p21 after pCDNA-MEG3 or empty vector transfection.** Results shown are from 3 independent experiments. GAPDH protein was used as an internal control. * *P* < 0.05; ** *P* < 0.01.

Further exploration of the mechanisms involved in *MEG3* overexpression induced growth arrest and apoptosis was done by examining the expression of p53 protein after transfection with pCDNA-MEG3 or empty vector. Recent studies have indicated that lncRNAs may play an important role in the regulation of cell growth by modulating p53 pathway [26]. The results of western blot analysis showed that the expression of p53 was significantly increased and the expression of MDM2 was downregulated in SPC-A1 cells transfected with pCDNA-MEG3 compared to those with empty vector. No significant differences were observed in the expression levels of p21 in SPC-A1 cells transfected with pCDNA-MEG3 compared to those with empty vector (Figure 6). These data confirm that *MEG3* functions as a tumor suppressor gene by regulating p53 activation in NSCLC.

## Discussion

Recently, genome-wide surveys have revealed that the human genome contains ~20000 protein-coding genes and >98% of the total genome can be transcribed, yielding many short or long noncoding RNAs (lncRNAs) with limited or no protein-coding capacity [27,28]. There are over 3000 human lncRNAs greater than 200nt in length, but less than 1% of them have been characterized [5,29]. Although only a minority have been characterized in detail, recent studies showed that lncRNAs participates in diverse biological processes including cell cycle control and cell differentiation through distinct mechanisms, such as imprinting, chromosome dosage-compensation, epigenetic regulation, mRNA splicing, nuclear and cytoplasmic trafficking [30-32]. Several studies have further demonstrated that lncRNAs are efficiently regulated during development in response to diverse signaling, and dysregulation of lncRNAs may also affect epigenetic information and provide a cellular growth advantage, resulting in progressive and uncontrolled tumor growth [10,16,33,34]. Although lncRNAs may have impact on human cancers, the basis of their molecular mechanisms is still not well known. Therefore, the interplay between proteins and lncRNAs is an important topic in the field of cancer biology, in which lncRNAs may provide the missing clue of the well-known oncogenic and tumor suppressor network.

To date, many lncRNAs have been identified, and their involvement in human cancer has been extensively reported. The lncRNA MALAT-1 expression was markedly increased in primary bladder tumors that subsequently showed evidence of metastasis, and its overexpression could promote bladder cancer cells invasion by modulating epithelial-mesenchymal transition (EMT)-associated ZEB1, ZEB2, Slug and E-cadherin levels or by activating Wnt signaling [35]. In this study, we found that the expression of lncRNA MEG3 was decreased in NSCLC tissues when compared to normal tissues. Specifically, *MEG3* expression was found to be significantly lower at later stages of tumor development and in tumors that had undergone increase in size. Moreover, the overall survival time of patients with moderate or strong *MEG3* expression levels was significantly higher than that of patients with lower *MEG3* expression levels. Moreover, loss or significant reduction of *MEG3* expression in various human primary tumors including neuroblastomas, hepatocellular cancers and gliomas has been well documented [21,36,37]. In addition, we demonstrate that *MEG3* expression is lost in multiple NSCLC cell lines compared to a normal human bronchial epithelial cell line (16HBE). Similarly, loss of *MEG3* expression has also been found in many cancer cell lines including those derived from brain, bladder, bone marrow, breast, cervix, colon, liver, lung, meninges and prostate [18]. We also showed that DNA methylation may underlie the lost expression of *MEG3* in NSCLC tissues. This suggests that the decreased expression of *MEG3* may be mediated by DNA methylation and useful in the development of novel prognostic or progression markers for NSCLC.

In order to highlight the impact of dysregulated expression and function of *MEG3*, we show the critical role of *MEG3* in the development of NSCLC. Ectopic expression of *MEG3* by transfection decreased the cell growth, and led to the promotion of cell apoptosis *in vitro* and *in vivo*. To further investigate how *MEG3* induces NSLCC cells apoposis and growth arrest, we examined the level of p53 after transfection of pCDNA-MEG3 in SPC-A1 cells. We found that re-expression of *MEG3* could significantly stimulate the level of p53 protein. Peng-jun Wang and Yunli Zhou have also reported that non-coding RNA MEG3 may function as a tumor suppressor mediated by inducing the activation of p53 [21,22]. As an important transcription factor, p53 is capable of regulating expression of many target genes leading to the suppression of tumor development and growth, and it is mutated in most human cancers [38]. Generally, p53 level is very low due to rapid degradation via the ubiquitin-proteasome pathway. The ubiquitination of p53 is mainly mediated by MDM2, an E3 ubiquitin ligase. Inhibition of MDM2 plays a major role in p53 stabilization. A decrease in MDM2 protein level was observed in SPC-A1 cells transfected with pCDNA-MEG3, suggesting that MDM2 downregulation is one of the mechanisms by which *MEG3* activates p53. Interestingly, the results revealed that *MEG3* does not stimulate p21^Cip1^ expression, a well-known p53 target gene. These findings indicate that lncRNA *MEG3* may function as a tumor suppressor by activating p53 and underlying target genes, but not p21^Cip1^, and its deficiency or decreased expression or function could contribute to NSCLC development.

## Conclusions

In summary, we demonstrate that the loss of lncRNA MEG3 expression is a common event underlying NSCLC, suggesting that *MEG3* may play a key functional role in NSCLC developmeng and as a negative prognostic factor for NSCLC patients and an indicative of poor survival rates. The current study provides novel role of lncRNAs, specifically *MEG3*, and may help us to better understand the pathogenesis and development of NSCLC. Further understanding of this mechanism will foster the development of lncRNA-directed diagnostic and therapeutic agents against NSCLC.

## Competing interests

The authors declare that they have no competing interests.

## Authors’ contributions

LKH, LW and XWP were involved in the conception and design of the study. ZML and WWQ was involved in the provision of study material and patients. LXH, SM and HYY performed the data analysis and interpretation. LKH wrote the manuscript. XWP approved the final version. All authors read and approved the final manuscript.

## Pre-publication history

The pre-publication history for this paper can be accessed here:

http://www.biomedcentral.com/1471-2407/13/461/prepub

## Supplementary Material

Additional file 1 **Clinical data, such as age, gender, TNM stage at.** Al of individual patients. Click here for file

Additional file 2 **Inhibition of *MEG3 *promotes cell proliferation in vitro. (A)** Analysis of MEG3 expression levels in H1299 cells transfected with si-MEG3 or si-NC by qRTPCR. **(B)** MTT assay was performed to determine the proliferation of H1299 cells. Data represent the mean ± S.D. from three independent experiments. **(C)** Colonyforming growth assays were performed to determine the proliferation of H1299 cells. The colonies were counted and captured. Click here for file
