# Supplementary figures and images for: Long non-coding RNA MEG3 inhibits NSCLC cells proliferation and induces apoptosis by affecting p53 expression
Source: BMC Cancer. 2013 Oct 7;13:461. doi: 10.1186/1471-2407-13-461 (PMC3851462; doi:10.1186/1471-2407-13-461)

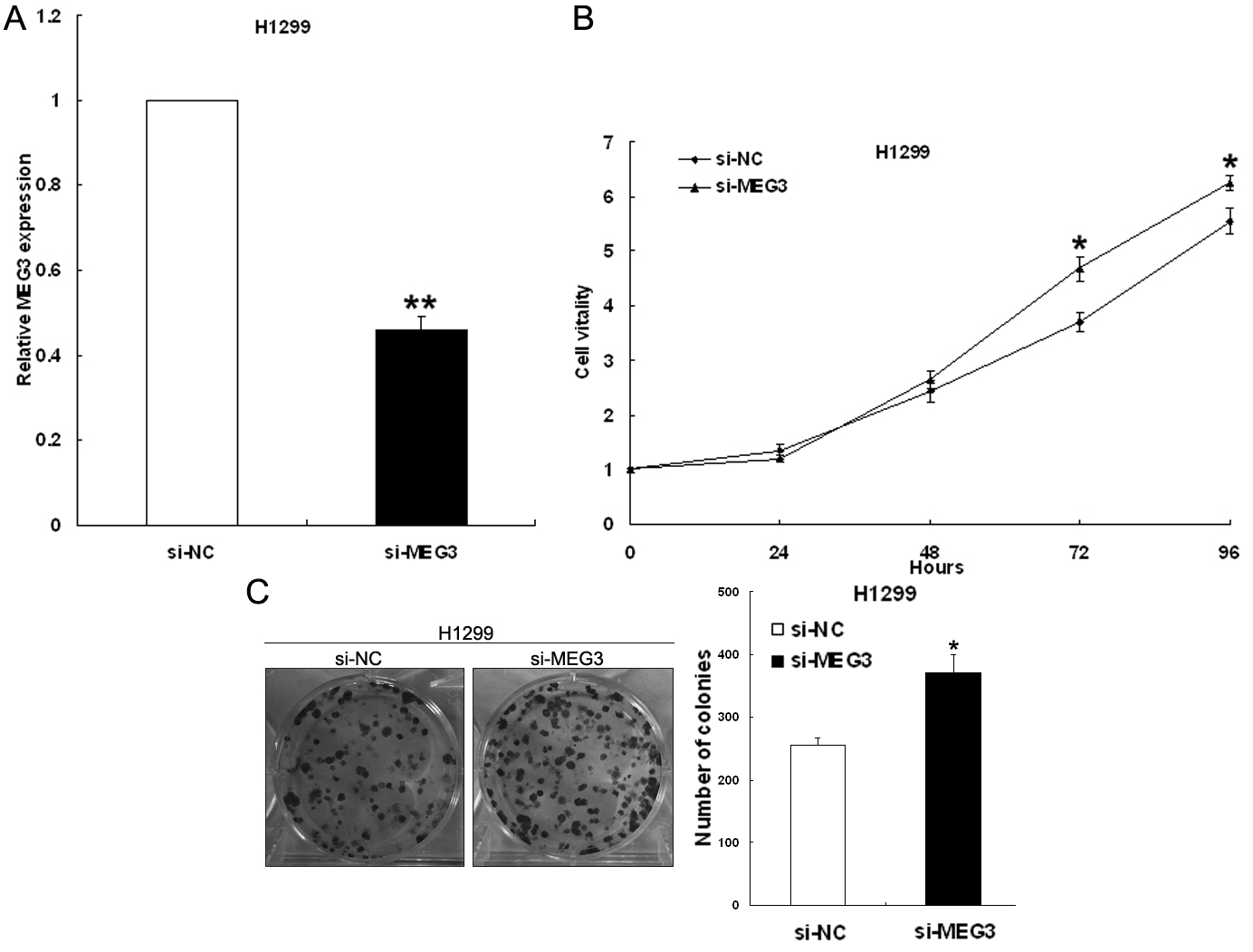

Supplement: Additional file 2 — Inhibition of MEG3 promotes cell proliferation in vitro. (A) Analysis of MEG3 expression levels in H1299 cells transfected with si-MEG3 or si-NC by qRTPCR. (B) MTT assay was performed to determine the proliferation of H1299 cells. Data represent the mean ± S.D. from three independent experiments. (C) Colonyforming growth assays were performed to determine the proliferation of H1299 cells. The colonies were counted and captured. [file 1471-2407-13-461-S2.tiff]
